# Health literacy correlates, barriers, and adherence in post-prostatectomy sexual rehabilitation: cross-sectional findings from China

**DOI:** 10.3389/fpubh.2026.1725144

**Published:** 2026-03-20

**Authors:** Lihan Wei, Ye Wu, Fei Wei, Yongxiang Yi

**Affiliations:** Urinary Surgery, The Third Affiliated Hospital of Zhejiang University of Traditional Chinese Medicine, Hangzhou, Zhejiang, China

**Keywords:** health literacy, knowledge-attitudes-practices, prostate cancer, radical prostatectomy, sexual health, treatment adherence

## Abstract

**Background:**

Sexual dysfunction following radical prostatectomy remains a persistent challenge affecting patient quality of life. Health literacy may influence engagement with multimodal rehabilitation strategies, yet its role in sexual health recovery practices remains poorly understood, particularly in non-Western populations.

**Methods:**

This single-center cross-sectional Knowledge-Attitudes-Practices survey was conducted at a tertiary urology center in China from March 2023 through June 2025. We enrolled 1,615 men following radical prostatectomy and collected partner-reported items where available, stratifying participants by health literacy tertiles, using the validated HLS19-Q12 instrument. Primary outcomes included knowledge scores, treatment utilization (PDE5 inhibitors, vacuum devices, injection therapy, pelvic floor training), multimodal engagement, and adherence rates. Statistical analyses employed hierarchical logistic regression, mediation analysis, and latent class modeling.

**Results:**

Among 1,615 men (low *n* = 538; moderate *n* = 539; high *n* = 538), higher (vs low) health literacy was associated with greater PDE5 inhibitor use (adjusted OR 1.42; 95% CI: 1.08–1.86; *p* = 0.01), injection therapy utilization (adjusted OR 1.58; 95% CI: 1.16–2.15; *p* = 0.004), multimodal engagement (≥2 modalities: adjusted OR 1.56; 95% CI: 1.18–2.06; *p* = 0.002), and adherence ≥80% (adjusted OR 1.71; 95% CI: 1.30–2.25; *p* < 0.001). Structural equation modeling indicated that health literacy was statistically associated with an indirect pathway consistent with partial mediation of the knowledge and practice association (proportion mediated 0.31; 95% CI: 0.18–0.49; *p* < 0.001). Treatment barriers decreased systematically: side effect concerns (44.1% vs. 28.1%, OR 0.51, *p* < 0.001), embarrassment (35.1% vs. 20.8%, OR 0.49, *p* < 0.001), and cost concerns (49.1% vs. 32.3%, OR 0.50, *p* < 0.001). Information-seeking behaviors increased dramatically with higher literacy: active health information seeking (35.1% vs. 66.2%, OR 3.64, *p* < 0.001) and digital resource use (27.0% vs. 61.2%, OR 4.27, *p* < 0.001). Latent class–based risk stratification identified a high-risk class comprising 22% of participants who may benefit from immediate intervention.

**Conclusion:**

Health literacy is a fundamental, modifiable correlation of post-prostatectomy sexual recovery, demonstrating statistical associations consistent with knowledge to practice mediation pathways. Cross-sectional findings reveal substantial literacy-stratified disparities in treatment utilization and adherence, warranting prospective trials of routine health literacy assessment and tiered intervention strategies to reduce inequities and improve sexual health outcomes.

## 1 Introduction

Prostate cancer (PCa) is a major and growing global cancer burden. GLOBOCAN 2022 estimates indicate that PCa contributes roughly 7% of incident cancers worldwide, and the number of men living with this cancer is projected to rise as populations age and survival improves (1, 2). Consequently, survivorship care has become central to oncology agendas, with international commissions calling for scalable supportive interventions that preserve quality of life (3). Radical prostatectomy (RP)—open, laparoscopic, or robotic—remains a cornerstone treatment for localized disease, yet erectile dysfunction (ED) and broader sexual dissatisfaction are frequent and persistent sequelae that impair patient and partner well-being (4–6). Across contemporary cohorts, rates of clinically meaningful ED after RP vary with patient selection, technique, and definitions but remain consistently high, positioning sexual health recovery as a priority in comprehensive survivorship programs (4, 6, 7).

Multiple rehabilitation modalities can support post-RP sexual recovery—phosphodiesterase-5 inhibitors (PDE5i), vacuum erection devices (VED), intra-cavernosal injections, structured pelvic floor muscle training (PFMT), and psychosexual counseling—yet implementation is heterogeneous and long-term adherence is often suboptimal (8, 9). Recent syntheses show that PDE5i improves erectile function during active use, although effects on spontaneous recovery are inconsistent; combination strategies (e.g., PDE5i plus VED) may help selected patients (7, 8, 10). VED-based programs and early, protocolized initiation have been associated with short-term benefits, but optimal schedules remain debated (8, 9). Beyond devices and pharmacotherapy, dyadic and psychosocial interventions address intimacy, communication, and treatment self-efficacy—determinants tightly linked to sustained practice of rehabilitation behaviors (10–12). Collectively, the literature suggests that multi-modal, behaviorally supported rehabilitation is most likely to generate meaningful recovery across diverse patient populations.

Health literacy—defined as the capacity to access, understand, appraise, and apply health information to make informed health decisions across healthcare, disease prevention, and health promotion domains (13), has emerged over the last decade as a fundamental determinant of cancer outcomes (12). However, limited HL is associated with poorer comprehension of options, lower perceived shared decision-making, reduced adherence, and worse quality of life across tumor types and care settings (14, 15). Within PCa, recent studies link lower HL to diminished participation in survivorship care and poorer patient-reported outcomes, whereas higher HL correlates with stronger self-efficacy and more active information seeking (16–18). The recent validation of brief HL instruments for Chinese adults facilitates routine HL assessment in Asian oncology contexts (19). Notably, HL may also modify the uptake and impact of couple-focused and digital interventions by shaping how patients and partners interpret guidance and translate it into daily practices (16, 20).

Sexual recovery following PCa is fundamentally a dyadic process, which has necessitated the development of couple-based psychoeducational interventions (20–22). However, meta-analytic evidence reveals that while these programs yield consistent psychosocial benefits, their impact on global quality-of-life metrics is less certain, with a paucity of large-scale trials in non-Western cohorts (23). The design of pragmatic, scalable interventions is therefore predicated on systematically mapping a knowledge, attitudes, and practices (KAP) regarding sexual rehabilitation, alongside the modulating role of HL (60). When integrated with HL assessment, this approach delineates knowledge deficits, perceived barriers, and real-world adherence to multimodal regimens, thereby providing an empirical basis for designing tiered, literacy-responsive interventions including scalable telehealth models. Importantly, while general health literacy encompasses foundational competencies in accessing and applying health information, eHealth literacy extends these skills to digital contexts—requiring additional proficiencies in navigating online platforms, evaluating web-based information quality, and engaging with interactive technologies. General HL thus serves as a prerequisite for effective eHealth engagement, and our findings regarding HL-stratified digital resource use suggest that telehealth implementations must scaffold both general and digital-specific literacy competencies (22, 24, 25).

China offers a distinctive context for this inquiry, with rising PCa incidence—part of approximately 4.8–4.9 million new cancer cases nationally in 2022, with recent increases reported in several provinces—driven by an aging population, risk factors, and expanded screening (26, 27). Despite the widespread use of robotic-assisted RP, Chinese studies consistently document high rates of sexual morbidity and substantial unmet informational and psychological needs for patients and their partners post-treatment (26, 28, 29). These issues are compounded by culture-specific barriers, such as stigma around sexual discourse and reluctance to disclose intimacy concerns, which impede rehabilitation engagement (29, 30, 61–63). Furthermore, a Chinese randomized study highlighting that combining modalities like PDE5i and VED optimizes outcomes underscores the critical need for an implementation-focused approach in routine practice (30).

From a health-systems perspective, China has invested in HL promotion and surveillance since 2012; nevertheless, the proportion of adults with adequate HL remains below one-third and is lowest among older and rural populations—groups disproportionately affected by PCa (30, 31). Chinese studies associate limited HL with lower self-efficacy, less favorable health behaviors, and greater medical expenditures, while concise, validated HL measures support clinical integration and research (30, 32, 33). Given these structural and cultural dynamics, HL likely shapes KAP profiles, thereby driving sexual recovery trajectories in China.

Despite global research on the efficacy of individual rehabilitation modalities, important gaps persist. Few studies link HL and dyadic factors to the implementation and adherence of multimodal sexual rehabilitation, and the mediation pathways through which HL converts knowledge into practice are rarely tested (22, 33–35). In China, quantitative KAP studies integrating HL to map these determinants are scarce, limiting evidence-based risk stratification and intervention tailoring (28–30, 34, 36). Therefore, the objectives of this study were fourfold: (1) to quantify knowledge, attitudes, and utilization patterns of core sexual rehabilitation modalities (PDE5i, VED, injection therapy, PFMT) among men following RP; (2) to examine the independent association between HL and sexual health recovery practices after adjusting for clinical and sociodemographic factors; (3) to test whether HL mediates the relationship between rehabilitation knowledge and practice implementation; and (4) to identify HL-stratified barriers and facilitators that may inform intervention design. Partner-reported data were collected to contextualize dyadic support constructs where applicable.

## 2 Methodology

### 2.1 Study design and setting

This was a single-center, observational, cross-sectional KAP survey conducted at a tertiary urology center in China from March 2023 through June 2025. The study focused on men after RP and their intimate partners. Data were collected cross-sectionally with concurrent abstraction of clinical variables from the electronic medical record; all questionnaires were administered in Chinese by trained research staff using tablet-based forms in private clinic rooms.

### 2.2 Sample size and precision

Recognizing that this observational study examines associations rather than testing a single null hypothesis, we adopted precision-based sample size estimation—an approach increasingly advocated for exploratory and association studies where traditional power calculations may be overly restrictive or poorly matched to analytical complexity (e.g., mediation models, latent class analysis). However, to provide transparency regarding detectable effect sizes, we calculated that with the achieved cohort of N = 1,615 men, two-sided *α* = 0.05, and 80% power, minimum detectable odds ratios for the association between health literacy (tertiles) and the primary composite implementation outcome (engagement with ≥2 modalities plus ≥80% adherence) were in the order of 1.4–1.6 across plausible baseline prevalences of 0.20–0.35 in the low-literacy stratum. Additionally, for the mediation analysis, this sample provided 80% power to detect indirect effects as small as 0.03 standardized units, and for latent class analysis, the sample exceeded recommended thresholds of 5–10 observations per estimated parameter. The target sample size also accommodated anticipated non-response and incomplete dyadic participation. During the recruitment period, 1,847 men were approached; 12 were excluded during screening for inability to complete self-administered questionnaires, leaving 1,835 eligible participants. Of these, 1,615 consented and completed surveys (response rate 88.0%). Among these, 1,203 (74.5%) had participating partners who completed partner-directed items, while 412 (25.5%) enrolled without partner participation. Non-participation was primarily due to partner unavailability at the time of clinic visit (*n* = 312, 75.7%) or partner declination (*n* = 100, 24.3%). The target sample was operationalized through consecutive sampling across clinics and time blocks.

### 2.3 Eligibility criteria

Inclusion criteria were age ≥18 years; receipt of RP for histologically confirmed prostate cancer; postoperative follow-up at the recruiting center ≥6 weeks post-surgery; and ability to read Chinese and complete self-administered questionnaires. Partner participation was not an eligibility criterion; however, men in intimate relationships were encouraged to invite their partners to complete partner-directed survey items to enrich dyadic construct measurement. Exclusion criteria were neurocognitive impairment precluding reliable self-report; salvage or palliative surgeries not intended for cure; and active adjuvant therapies at the time of survey that would preclude attribution of sexual function to the postoperative state.

Ability to complete self-administered questionnaires was assessed by research coordinators using a standardized 3-item screen: (1) Can you read this consent form without assistance? (2) Can you explain in your own words what this study is asking you to do? (3) Do you feel able to answer questions about your health and treatments on a tablet? Participants answering ‘no’ to any item were excluded (n = 12 during screening, representing 0.6% of approached individuals).

### 2.4 Participants and recruitment

Eligible participants were consecutive adult men who had undergone RP (open, laparoscopic, or robotic) for localized prostate cancer and were attending routine postoperative follow-up at any scheduled visit ≥6 weeks post-surgery (median time at enrollment: 18 months post-surgery, IQR 9–36 months). This timeframe ensured that all participants had been counseled on rehabilitation options at their initial post-operative visit and had sufficient opportunity to engage with modalities, while capturing variation in recovery trajectories from early post-operative to long-term survivorship phases. Each partnered participant was invited to nominate an intimate partner (spouse/long-term partner) to complete partner-directed items assessing perceived partner support, relationship satisfaction, and communication patterns. Men without current intimate partnerships (n = 179, 11.1%) were enrolled and completed all patient-directed items; partner-specific constructs were coded as missing for these individuals. Recruitment used consecutive sampling across urology clinics and postoperative counseling sessions. Eligible patients were identified by clinic nurses during registration and referred to trained research coordinators (*n* = 3, all with backgrounds in urology nursing) who verified eligibility, explained the study, and obtained written informed consent. Research coordinators also approached partners when present to invite dyadic participation. Written informed consent was obtained from both members of the dyad prior to any data collection.

Standard care followed institutional protocols with assessments at 4–6 weeks, 3, 6, 12, and 24 months. Management included urologist-led verbal counseling on rehabilitation (PDE5i, VED, injections, PFMT) and prescriptions based on preference and nerve-sparing status. Optional resources included written handouts, nurse-led device training, and psychosexual referrals (uptake <15%). Routine care excluded systematic health literacy screening or mandatory training; the study survey was observational and did not alter clinical management.

### 2.5 Data collection procedures

After eligibility confirmation, research staff administered the KAP survey and HL instrument to patients in private clinic rooms. Partners who accompanied patients to the visit were separately consented and completed partner-directed items concurrently on separate tablets in adjacent private spaces. No remote or delayed partner recruitment was attempted; partners not present at the index clinic visit were not subsequently contacted, representing a pragmatic design aligned with routine care workflows. Clinical variables—including surgical approach, nerve-sparing status, and time since surgery—were abstracted from the medical record using a standardized template. All responses were time-stamped to permit calculation of “time since surgery” in months. Data quality procedures included programmed range checks, logic checks (e.g., mutually exclusive response options), and a double-entry verification of 10% of records.

### 2.6 Bias mitigation strategies

Several design and fieldwork procedures were implemented to limit bias. To reduce recall bias, modality-specific utilization was captured within structured windows (28 days for PDE5i and VED; 90 days for injection therapy), and adherence questions were anchored to prescribed schedules (8, 9, 30, 34). Selection bias was mitigated through consecutive sampling across multiple clinics and time periods. To minimize social-desirability and partner-influence biases, surveys were self-administered on tablets in private rooms using neutral, standardized prompts; staff intervened only to clarify wording without leading content or offering advice.

### 2.7 Measures

#### 2.7.1 Sociodemographic characteristics

Patients reported age (continuous, years), marital status (married vs. single/divorced/widowed), and highest educational attainment categorized as primary or below, junior secondary, senior secondary, technical college, and university/postgraduate. These categories were used for descriptive stratification and modeling, with university/postgraduate serving as the reference in multivariable analyses where specified by the tables.

#### 2.7.2 Clinical characteristics

Time since surgery (months) was derived from the date of RP to the survey date and summarized as median (IQR) for between-group comparisons. Nerve-sparing status was classified as bilateral, unilateral, or none. Surgical approach was categorized as robotic, laparoscopic, or open. Preoperative erectile function (as documented by the treating team in clinic notes) was included as an adjustment variable in models examining treatment utilization and adherence.

#### 2.7.3 Health literacy

Health literacy was assessed using the HLS19-Q12 (Chinese version), a validated 12-item instrument that measures the perceived ease or difficulty of finding, understanding, appraising, and applying health information across healthcare, disease prevention, and health promotion domains (19). Items were scored on a four-point Likert scale and transformed per the scoring manual to a standardized index (range 0–50), with higher values indicating greater literacy. Internal consistency in this cohort was excellent (Cronbach’s *α* = 0.87). For descriptive and inferential analyses, the index was partitioned into tertiles to define Low (*n* = 538, HLS19-Q12 score 0–33), Moderate (*n* = 539, score 34–41), and High (*n* = 538, score 42–50) literacy groups, which served as the primary exposure. These sample-derived tertile cut-points ensured equal group sizes for statistical power while preserving dose–response assessment; exact thresholds will vary across populations with different literacy distributions. Tertile categorization was chosen for several methodological and clinical reasons: (1) it maximizes statistical power for regression analyses by creating relatively equal-sized groups while preserving dose–response assessment; (2) it avoids reliance on externally derived cut-points (e.g., “adequate” vs. “limited” literacy) that may not be validated or culturally appropriate for Chinese populations; (3) it facilitates clinical translation by stratifying the local patient population into meaningful risk tiers rather than imposing arbitrary absolute thresholds; and (4) sensitivity analyses treating health literacy as a continuous variable yielded consistent effect estimates, supporting robustness to categorization approach. However, we acknowledge that tertile cut-points are sample-dependent and may not generalize to populations with markedly different literacy distributions.

#### 2.7.4 Knowledge assessment

Knowledge was measured using a 12-item prostate-cancer rehabilitation knowledge questionnaire (score range 0–12, 1 point per correct response). Items covered (i) expected recovery timelines after nerve-sparing vs. non-sparing RP, (ii) impact of androgen-deprivation therapy on erectile function, (iii) role and limitations of PDE5 inhibitors (PDE5i), (iv) appropriate use and safety of VED, (v) benefits of PFMT, and (vi) general safety considerations for rehabilitation. The total knowledge score (continuous) served as an outcome for regression modeling and as an input for latent class analysis; item content was aligned with contemporary rehabilitation guidance and empirical literature on PDE5i, VED, and scheduled combination strategies in post-prostatectomy care (8, 9, 30, 34).

#### 2.7.5 Attitudes and beliefs

Attitudinal constructs were assessed on five-point Likert scales (1 = strongly disagree to 5 = strongly agree) and scored as the mean of constituent items, with higher values indicating greater endorsement of the underlying construct. Domains comprised treatment acceptability for VED and for intracavernosal injection therapy; self-efficacy for practicing rehabilitation behaviors alongside confidence in recovery; perceived barriers encompassing side-effect concerns, embarrassment, cost barriers, and privacy concerns; and perceptions of support and healthcare delivery including partner-support importance, telehealth convenience, and trust in clinicians. Scale reliability was evaluated during instrument piloting using internal consistency metrics, and composite scores were entered as continuous covariates in multivariable models; attitudinal domains also reflected constructs emphasized in contemporary dyadic and telehealth rehabilitation programs (12, 37).

Specifically, the ‘partner support importance’ construct incorporated both patient self-report (perceived partner support) and partner-completed items (partner-reported willingness to engage in rehabilitation activities; partner comfort discussing sexual concerns). These were averaged to create a composite dyadic support score for the 1,203 dyads (74.5%) with complete data from both members; for the remaining 412 patients (25.5%) without participating partners, only patient-reported perceived support was available. All other attitudinal domains reflected patient self-report only.

#### 2.7.6 Sexual health recovery practices

Practice variables indexed real-world use and adherence to rehabilitation modalities in prespecified recall windows. Phosphodiesterase-5 inhibitor (PDE5i) use was defined as any patient-reported intake within the prior 28 days; VED therapy as any use within the prior 28 days; and injection therapy as any intra-cavernosal injection within the prior 90 days. PFMT engagement reflected active participation in a structured program at the time of survey. Multimodal engagement was summarized with binary indicators for the use of at least two modalities and of at least three modalities among PDE5i, VED, injection therapy, and PFMT. Rehabilitation support utilization captured whether a written rehabilitation plan had been provided by clinicians and whether in-clinic training for device or injection use had been received. Adherence was operationalized as good adherence (≥80%) when respondents reported completing at least 80% of the prescribed frequency or dosing across all modalities currently in use during the relevant observation windows, measured through structured frequency items anchored to each modality’s prescription schedule.

#### 2.7.7 Information-seeking behaviors

Information-seeking behaviors were recorded as binary indicators capturing active health-information search (for example, reading educational materials or posing targeted questions to clinicians), use of digital health resources (including hospital portals, vetted medical websites, or telehealth applications), and participation in in-person or virtual support groups. These indicators were summarized descriptively and examined in literacy-stratified models.

#### 2.7.8 Derived composite outcomes and profiles

The primary composite outcome, termed optimal sexual health recovery practices, was defined as concurrent engagement with at least two rehabilitation modalities together with achievement of good adherence (≥80%). A practice engagement score (range 0–6) was derived by summing binary modality indicators (PDE5i, VED, injection therapy, and PFMT; 1 point each) with support utilization markers (written plan and in-clinic training; 1 point each), providing a continuous index for exploratory profiling. Latent class analysis (LCA) used discrete categories of health literacy tertile, knowledge (Low <6; Moderate 6–8; High >8), and practice (Poor <2; Moderate 2–3; Good >3, based on the practice engagement score) to identify phenotypes, which were subsequently mapped to intervention priorities (Priority 1–3 and Mixed). Patterns of missingness were profiled by variable and domain and evaluated using Little’s MCAR test (χ^2^ = 127.4, df = 143, *p* = 0.81, supporting MCAR assumption). For the partner support construct (Tables 1, 2), which incorporated both patient-perceived and partner-reported support, two analytic approaches were employed: (1) primary analyses used composite dyadic support scores for complete dyads (*n* = 1,203) and patient-perceived support only for patients without participating partners (*n* = 412), treating the construct as a single variable with internally consistent measurement properties (ICC = 0.71 between patient-perceived and partner-reported support); (2) Sensitivity analyses restricted to complete dyads yielded nearly identical health literacy associations: for strong partner support, OR = 1.69 (95% CI: 1.21–2.36) in complete dyads versus OR = 1.72 (1.24–2.39) in the full sample; for the primary composite outcome (Table 3), high vs. low literacy OR = 1.91 (1.39–2.61) in complete dyads versus OR = 1.89 (1.41–2.53) in the full sample. These findings indicate that partner participation enriched measurement precision (ICC = 0.71 between patient-perceived and partner-reported support) but did not materially alter the magnitude or direction of literacy-practice associations. All other outcomes (knowledge, treatment utilization, adherence, barriers) reflected patient self-report only and were thus unaffected by partner participation status. As a contingency, multiple imputation by chained equations (including all model covariates and outcomes) were prespecified under MAR assumptions (9).

**Table 1:** Attitudes toward sexual health recovery interventions by patient characteristics.

Attitude domain	Overall Mean (SD)	Low health literacy Mean (SD)	Moderate health literacy Mean (SD)	High health literacy Mean (SD)	*F* statistic	*P-*value
Treatment acceptability (1–5 scale)^a^						
VED acceptability	3.6 (1.1)	3.4 (1.2)	3.6 (1.1)	3.8 (1.0)	8.42	<0.001
Injection acceptability	3.2 (1.3)	2.9 (1.4)	3.2 (1.3)	3.5 (1.2)	12.67	<0.001
Self-efficacy and confidence						
Treatment self-efficacy	3.5 (1.1)	3.2 (1.2)	3.5 (1.1)	3.8 (1.0)	15.23	<0.001
Confidence in recovery	3.4 (1.2)	3.1 (1.3)	3.4 (1.2)	3.7 (1.1)	11.89	<0.001
Barriers to treatment						
Side effect concerns	3.3 (1.1)	3.5 (1.1)	3.3 (1.1)	3.1 (1.1)	7.94	<0.001
Embarrassment	2.8 (1.2)	3.0 (1.3)	2.8 (1.2)	2.6 (1.1)	6.47	0.002
Cost barriers	3.4 (1.2)	3.7 (1.1)	3.4 (1.2)	3.1 (1.2)	13.42	<0.001
Support and healthcare delivery						
Partner support importance	4.1 (0.9)	4.0 (1.0)	4.1 (0.9)	4.2 (0.8)	3.78	0.02
Telehealth convenience	4.0 (1.0)	3.8 (1.1)	4.0 (1.0)	4.2 (0.9)	9.15	<0.001
Trust in clinicians	3.8 (1.0)	3.7 (1.1)	3.8 (1.0)	3.9 (0.9)	2.94	0.05

^a^ Scale: 1 = strongly disagree, 2 = disagree, 3 = neither agree nor disagree, 4 = agree, 5 = strongly agree. Statistical Analysis: One-way ANOVA to examine interaction effects between health literacy and clinical factors on attitude scores.

**Table 2:** Health literacy-stratified analysis of barriers and facilitators to treatment adherence.

Barrier/Facilitator	Low health literacy (*n* = 538) No. (%)	Moderate health literacy (*n* = 539) No. (%)	High health literacy (*n* = 538) No. (%)	OR (95% CI)^a^	*P* for interaction
Treatment barriers					
High side effect concern	237 (44.1)	194 (36.0)	151 (28.1)	0.51 (0.39–0.67)	<0.001
Significant embarrassment	189 (35.1)	151 (28.0)	112 (20.8)	0.49 (0.36–0.67)	<0.001
High cost concern	264 (49.1)	219 (40.6)	174 (32.3)	0.50 (0.38–0.65)	<0.001
Privacy concerns	156 (29.0)	118 (21.9)	89 (16.5)	0.48 (0.34–0.68)	0.002
Treatment facilitators					
Strong partner support	367 (68.2)	398 (73.8)	423 (78.6)	1.72 (1.24–2.39)	0.01
High treatment self-efficacy	243 (45.2)	289 (53.6)	348 (64.7)	2.23 (1.69–2.95)	<0.001
Trust in healthcare providers	312 (58.0)	341 (63.3)	378 (70.3)	1.71 (1.26–2.32)	0.005
Telehealth acceptance	345 (64.1)	367 (68.1)	407 (75.7)	1.73 (1.26–2.37)	0.01
Information-seeking behaviors					
Actively seeks health information	189 (35.1)	259 (48.1)	356 (66.2)	3.64 (2.74–4.84)	<0.001
Uses digital health resources	145 (27.0)	221 (41.0)	329 (61.2)	4.27 (3.15–5.78)	<0.001
Participates in support groups	86 (16.0)	129 (23.9)	194 (36.1)	2.95 (2.12–4.11)	<0.001

OR indicates odds ratio; CI, confidence interval. ^a^ Odds ratio for high vs. low health literacy, adjusted for age, education, and clinical factors. Statistical Analysis: Multinomial logistic regression to identify differential barrier patterns across health literacy levels, with interaction terms.

**Table 3:** Multivariable analysis of factors associated with optimal sexual health recovery practices^a^.

Variable	Model 1 OR (95% CI)	Model 2 OR (95% CI)	Model 3 OR (95% CI)	*P-*value^b^
Demographics and clinical factors				
Age (per 10-y increase)	0.85 (0.74–0.98)	0.87 (0.75–1.00)	0.89 (0.77–1.03)	*0.12*
University education (vs primary)	1.67 (1.15–2.43)	1.45 (0.99–2.13)	1.28 (0.86–1.90)	*0.22*
Bilateral nerve-sparing (vs none)	2.34 (1.78–3.08)	2.28 (1.73–3.01)	2.25 (1.70–2.97)	*<0.001*
Time since surgery (>12 mo)	1.23 (0.95–1.60)	1.19 (0.91–1.55)	1.16 (0.89–1.52)	*0.27*
Knowledge (Model 2)				
Knowledge score (per 1-point increase)	—	1.18 (1.12–1.25)	1.14 (1.07–1.21)	*<0.001*
Attitudes (Model 2)				
Self-efficacy score (per 1-point increase)	—	1.42 (1.24–1.63)	1.35 (1.17–1.55)	*<0.001*
Low side effect concern (vs high)	—	1.56 (1.19–2.04)	1.48 (1.12–1.95)	*0.005*
Health literacy (Model 3)				
Moderate health literacy (vs low)	—	—	1.34 (1.01–1.78)	*0.04*
High health literacy (vs low)	—	—	1.89 (1.41–2.53)	*<0.001*
Model performance				
Nagelkerke *R*^2^	0.142	0.247	0.289	—
AIC	2134.5	1987.3	1952.8	—
C-statistic	0.681	0.743	0.768	—

OR indicates odds ratio; CI, confidence interval; AIC, Akaike information criterion. ^a^ Optimal sexual health recovery practices defined as engagement with ≥2 treatment modalities plus good adherence (≥80%). ^b^*P-*values from final model (Model 3). Statistical Analysis: Hierarchical logistic regression with sequential addition of knowledge, attitudes, and health literacy blocks to assess incremental predictive value.

### 2.8 Statistical analysis

Analyses followed a prespecified plan. Descriptive statistics summarized sociodemographic, clinical, literacy, knowledge, attitude, and practice variables; between-group differences across literacy tertiles were tested using ANOVA (or Kruskal–Wallis for skewed variables, e.g., months since surgery) and χ^2^ tests for categorical variables (two-sided *α* = 0.05). Determinants of knowledge (0–12) were estimated using multivariable linear regression with age group, education, marital status, time since surgery, and health-literacy tertile as covariates. Implementation outcomes were modeled via hierarchical logistic regression: Model 1 included clinical/sociodemographic covariates (age per 10 years, university education vs. primary, nerve-sparing status, time since surgery, with marital status and surgical approach as appropriate); Model 2 added knowledge, self-efficacy, and side-effect concern; Model 3 added health-literacy tertile. Effect sizes are reported as odds ratios (ORs) with 95% confidence intervals, alongside Nagelkerke *R*^2^, AIC, and C-statistics for model performance. Mediation analysis used structural equation modeling with standardized paths and bias-corrected bootstrap (5,000 resamples). The mediation model assumed linear relationships among continuous variables (knowledge score, health literacy index, practice engagement score) and normally distributed residuals; diagnostic plots confirmed adequate approximation of these assumptions. Model identification was verified through degrees of freedom calculations and rank checks. Indirect effects were estimated using the product-of-coefficients approach (Sobel test) and bias-corrected bootstrap confidence intervals, with statistical significance defined as 95% CI excluding zero.

Latent class analysis incorporated literacy tertile, knowledge (<6, 6–8, >8), and practice (<2, 2–3, >3) categories. LCA assumed conditional independence of indicators within classes (i.e., that health literacy, knowledge, and practice are independent conditional on latent class membership) and local homogeneity (no residual within-class heterogeneity). Model fit was evaluated using the Bayesian Information Criterion (BIC), Akaike Information Criterion (AIC), and entropy (classification accuracy), with lower BIC/AIC and entropy >0.80 supporting model adequacy. The optimal 4-class solution (BIC = 8,347, AIC = 8,289, entropy = 0.84) was selected over 3-class (BIC = 8,512, entropy = 0.79) and 5-class (BIC = 8,401, entropy = 0.81) alternatives. Bootstrap likelihood ratio tests (*n* = 500 resamples) confirmed that the 4-class model provided significantly better fit than nested alternatives (*p* < 0.001). Predictive validity was appraised using ROC AUC against the composite implementation outcome, with class-specific predicted probabilities serving as the classification variable. Sensitivity analyses included re-specifying the endpoint as engagement with ≥3 modalities and excluding men surveyed <6 months post-surgery. Primary analyses used available-case estimation without imputation.

## 3 Results

### 3.1 Participant characteristics and health literacy distribution

The study cohort included 1,615 men post-RP, distributed across low (*n* = 538), moderate (*n* = 539), and high (*n* = 538) health literacy tertiles (Table 4). Compared to the high-literacy group, participants with low health literacy were significantly older (mean age 67.1 ± 8.9 vs. 63.4 ± 7.8 years, *p* < 0.001) and had markedly different educational backgrounds (*p* < 0.001), with more having only primary education or less (16.5% vs. 5.8%) and fewer having university/postgraduate degrees (8.6% vs. 33.5%). Clinically, the low-literacy group also had a longer median time since surgery (20 vs. 16 months, *p* = 0.04) and were less likely to have undergone robotic-assisted surgery (53.9% vs. 59.1%, *p* = 0.03), with correspondingly higher proportions receiving laparoscopic (28.8% vs. 25.8%) or open surgery (13.4% vs. 9.5%).

**Table 4:** Baseline characteristics of study participants by health literacy level (*N* = 1,615).

Characteristic	Overall (*N* = 1,615)	Low health literacy (*n* = 538)	Moderate health literacy (*n* = 539)	High health literacy (*n* = 538)	*P-*value
Demographic characteristics					
Age, mean (SD), y	65.2 (8.4)	67.1 (8.9)	65.0 (8.2)	63.4 (7.8)	<0.001
Relationship status, No. (%)					0.02
Married	1,436 (88.9)	472 (87.7)	481 (89.2)	483 (89.8)	
Single/divorced/widowed	179 (11.1)	66 (12.3)	58 (10.8)	55 (10.2)	
Education level, No. (%)					<0.001
Primary or below	172 (10.7)	89 (16.5)	52 (9.6)	31 (5.8)	
Junior secondary	396 (24.5)	165 (30.7)	134 (24.9)	97 (18.0)	
Senior secondary	445 (27.6)	156 (29.0)	156 (28.9)	133 (24.7)	
Technical college	275 (17.0)	82 (15.2)	96 (17.8)	97 (18.0)	
University/postgraduate	327 (20.2)	46 (8.6)	101 (18.7)	180 (33.5)	
Clinical characteristics					
Months since surgery, median (IQR)	18 (9–36)	20 (10–38)	18 (9–35)	16 (8–33)	0.04
Nerve-sparing procedure, No. (%)					0.18
Bilateral	838 (51.9)	270 (50.2)	284 (52.7)	284 (52.8)	
Unilateral	280 (17.3)	95 (17.7)	92 (17.1)	93 (17.3)	
None	428 (26.5)	150 (27.9)	137 (25.4)	141 (26.2)	
Not documented/unknown	69 (4.3)	–	–	–	
Surgical approach, No. (%)					0.03
Robotic	916 (56.7)	290 (53.9)	308 (57.1)	318 (59.1)	
Laparoscopic	437 (27.1)	155 (28.8)	143 (26.5)	139 (25.8)	
Open	187 (11.6)	72 (13.4)	64 (11.9)	51 (9.5)	
Not documented/unknown	75 (4.6)	–	–	–	

Data are presented as No. (%) unless otherwise indicated. IQR indicates interquartile range. ^a^ *p*-values calculated using χ^2^ tests for categorical variables and analysis of variance for continuous variables. Statistical Analysis: Chi-square tests for categorical variables and ANOVA for continuous variables to examine differences across health literacy tertiles.

Partner participation was 74.5% (*n* = 1,203 dyads; 412 patients enrolled without partners). Partners’ mean age was 61.3 ± 8.1 years; 92.1% were female. Partner health literacy (HLS19-Q12) correlated moderately with patient literacy (Spearman *ρ* = 0.34, *p* < 0.001). Partner non-participation did not differ by patient literacy tertile (χ^2^ = 2.14, *p* = 0.34), age (χ^2^ = 3.87, *p* = 0.14), or education (χ^2^ = 5.21, *p* = 0.27), supporting missing-completely-at-random assumptions. However, partnered status differed by literacy: low-literacy patients were more likely unpartnered (12.3% vs. 10.2% high-literacy, *p* = 0.02).

### 3.2 Knowledge assessment and associated factors

**Figure 1:**
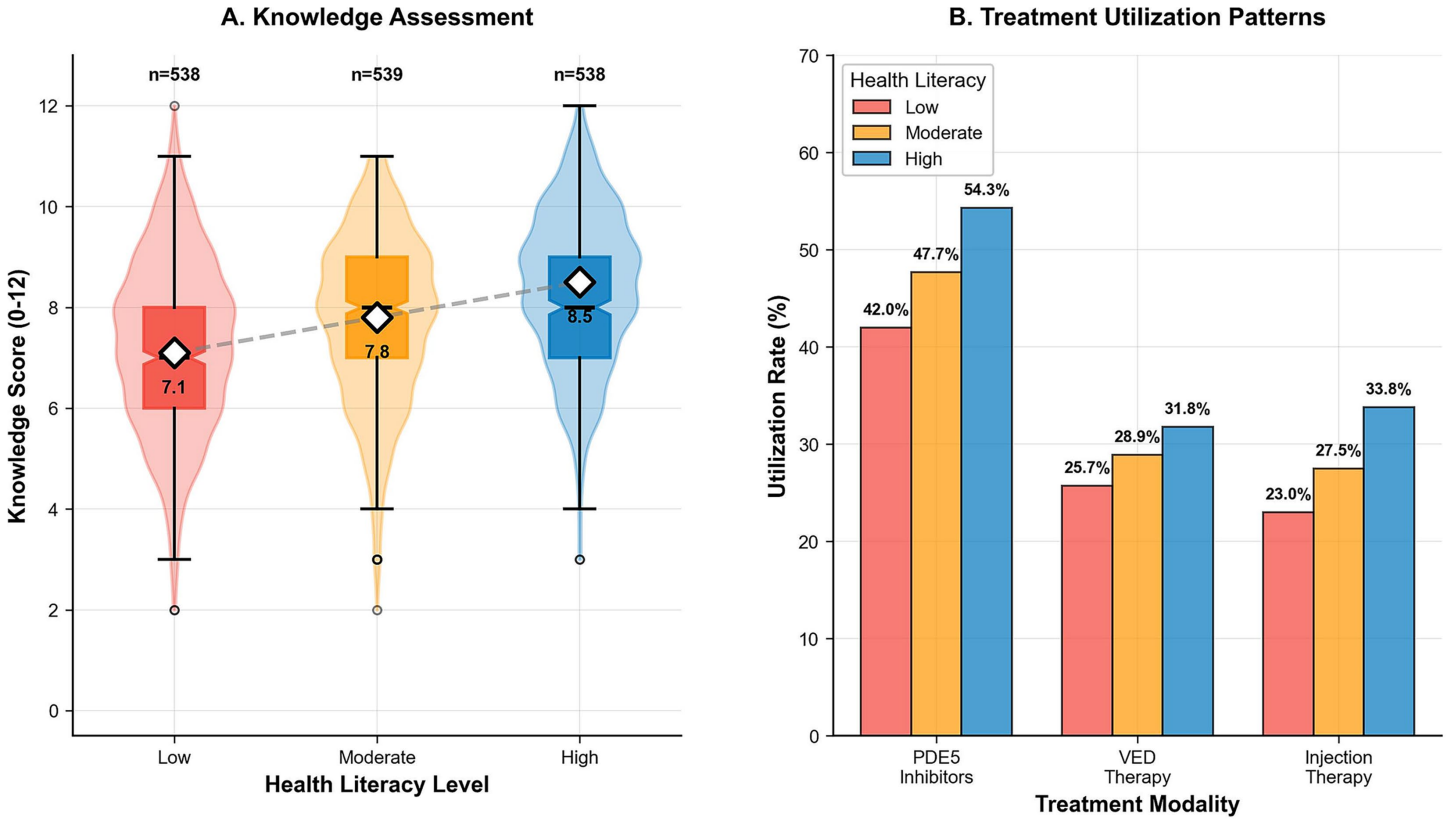
Knowledge and treatment utilization patterns by health literacy level. **(A)** Violin plots showing the distribution of rehabilitation knowledge scores across health literacy tertiles; embedded box plots denote medians and interquartile ranges, while the dashed line highlights the positive dose–response relationship. **(B)** Clustered bar chart displaying utilization rates for PDE5 inhibitors, VED, and injection therapy; consistent gradients across literacy levels illustrate systematic disparities in engagement with pharmacologic, device-based, and invasive modalities.

Health literacy demonstrated a strong dose–response relationship with rehabilitation knowledge scores (Table 5 and Figure 1A). Mean knowledge scores increased significantly across health literacy levels: 7.1 ± 2.4 for low, 7.8 ± 2.2 for moderate, and 8.5 ± 2.1 for high health literacy (all comparisons *p* ≤ 0.001). In multivariable analysis adjusting for demographics and clinical factors, moderate health literacy was associated with a 0.5-point increase in knowledge scores (95% CI: 0.2–0.8, *p* = 0.001), while high health literacy showed a 0.9-point increase (95% CI: 0.6–1.2, *p* < 0.001) compared to low health literacy. Additional independent predictors included education level, with university/postgraduate education associated with a 1.4-point increase versus primary education (95% CI: 0.9–1.9, *p* < 0.001), and age, with participants ≥70 years scoring 0.7 points lower than those <60 years (95% CI: −1.0 to −0.3, *p* < 0.001).

**Table 5:** Knowledge assessment scores by sociodemographic and clinical characteristics.

Characteristic	*n*	Knowledge score^a^ Mean (SD)	95% CI	Adjusted *β* (95% CI)^b^	*P-*value
Age Group, y					
<60	312	8.4 (2.1)	8.2–8.7	Reference	—
60–69	742	7.9 (2.3)	7.7–8.1	−0.3 (−0.6 to 0.0)	0.06
≥70	561	7.4 (2.4)	7.2–7.6	−0.7 (−1.0 to −0.3)	<0.001
Education level					
Primary or below	172	6.8 (2.5)	6.4–7.2	Reference	—
Secondary	841	7.7 (2.3)	7.5–7.9	0.6 (0.2 to 1.0)	0.005
University/postgraduate	327	8.9 (1.9)	8.7–9.1	1.4 (0.9 to 1.9)	<0.001
Time since surgery					
<12 months	485	7.6 (2.4)	7.4–7.8	Reference	—
12–24 months	394	8.0 (2.2)	7.8–8.2	0.3 (0.0 to 0.6)	0.04
>24 months	736	7.9 (2.3)	7.7–8.1	0.2 (−0.1 to 0.5)	0.15
Health literacy level					
Low	538	7.1 (2.4)	6.9–7.3	Reference	—
Moderate	539	7.8 (2.2)	7.6–8.0	0.5 (0.2 to 0.8)	0.001
High	538	8.5 (2.1)	8.3–8.7	0.9 (0.6 to 1.2)	<0.001

CI indicates confidence interval. ^a^ Knowledge score range: 0–12, with higher scores indicating better knowledge. ^b^ Adjusted for age, education, marital status, employment, time since surgery, and health literacy level. Statistical Analysis: Multivariable linear regression to identify independent predictors of knowledge scores, adjusted for confounders.

### 3.3 Sexual health recovery practices by health literacy

**Figure 2:**
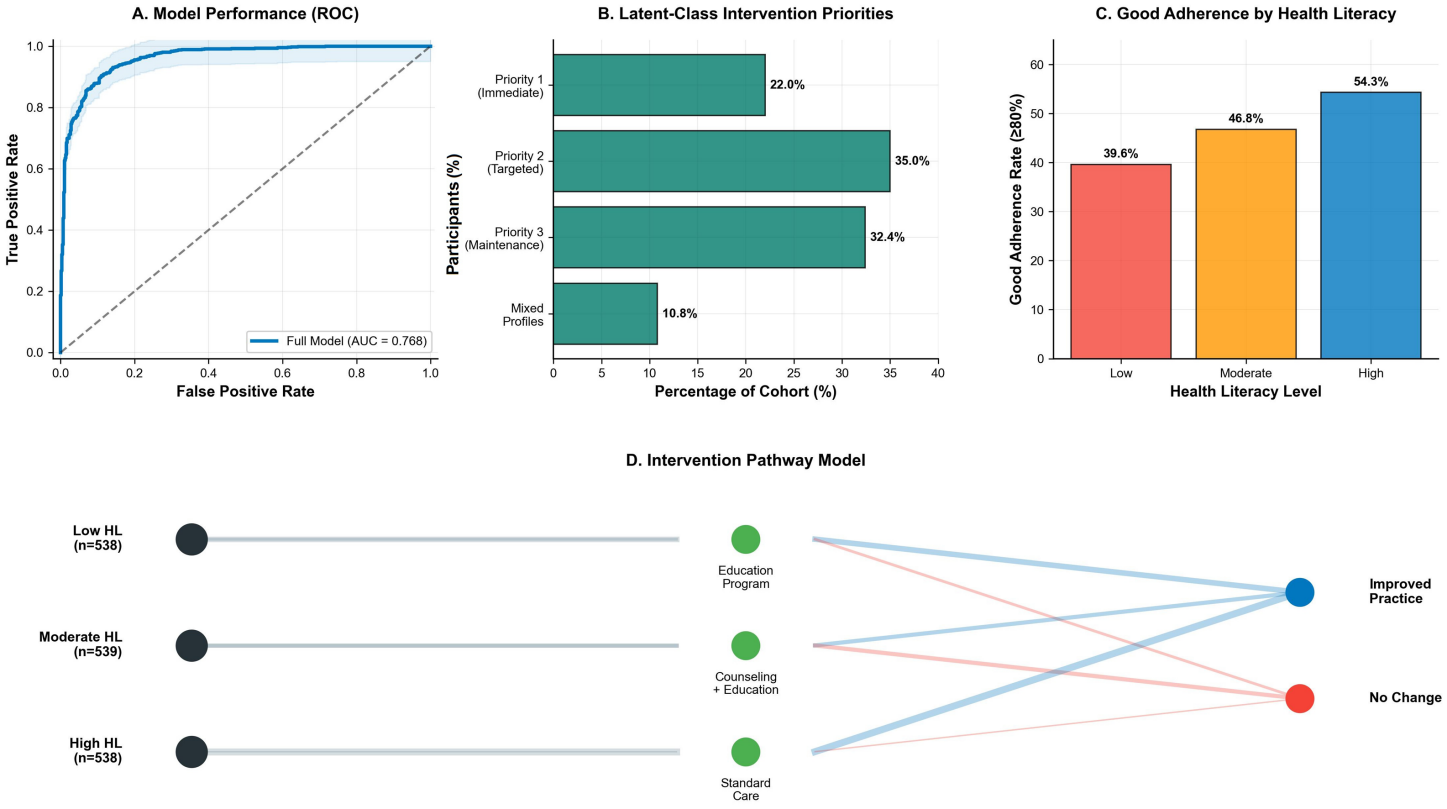
Clinical decision support and risk stratification framework. **(A)** ROC curve validating the risk stratification model’s discriminative ability to predict optimal rehabilitation engagement. **(B)** Cohort distribution across intervention priorities derived from latent class analysis: Priority 1 (Immediate) comprises high-risk patients requiring intensive support; Priority 2 (Targeted) and Priority 3 (Maintenance) represent intermediate and low-risk groups, respectively. **(C)** Dose–response gradient in adherence rates across health literacy levels; error bars represent 95% confidence intervals. **(D)** Conceptual framework for literacy-stratified rehabilitation. Patients are triaged by health literacy level (low, moderate, high) into matched intervention tiers (green nodes): intensive education with skills training (low HL), targeted counseling plus simplified materials (moderate HL), or standard care with digital resources (high HL). Blue lines denote primary pathways to successful engagement; red lines indicate non-adherence risk without adequate support.

Treatment utilization and adherence demonstrated consistent gradients across health literacy levels for all modalities (Table 6 and Figure 1B). From low to high literacy participants, utilization increased for PDE5 inhibitors (42.0% vs. 54.3%; adjusted OR 1.42, 95% CI: 1.08–1.86, *p* = 0.01), injection therapy (23.0% vs. 33.8%; adjusted OR 1.58, 95% CI: 1.16–2.15, *p* = 0.004), PFMT had the strongest association (7.1% vs. 11.2%; adjusted OR 1.73, 95% CI: 1.08–2.77, *p* = 0.02). Substantial disparities were also evident in multi-modal engagement, with the use of ≥2 modalities rising from 30.1 to 42.2% (adjusted OR 1.56, 95% CI: 1.18–2.06, *p* = 0.002) and ≥3 modalities showing an even more pronounced difference (8.9% vs. 15.1%; adjusted OR 1.89, 95% CI: 1.24–2.88, *p* = 0.003). The most striking disparity occurred in treatment adherence (≥80%), achieved by 39.6% of low-literacy versus 54.3% of high-literacy patients (adjusted OR 1.71; 95% CI: 1.30–2.25; *p* < 0.001) (Figure 2C).

**Table 6:** Sexual health recovery practices by health literacy level.

Treatment practice	Overall (*N* = 1,615)	Low health literacy (*n* = 538)	Moderate health literacy (*n* = 539)	High health literacy (*n* = 538)	Adjusted OR (95% CI)^a^	*P-*value
Treatment utilization, No. (%)						
PDE5 inhibitor use (past 4 weeks)	775 (48.0)	226 (42.0)	257 (47.7)	292 (54.3)	1.42 (1.08–1.86)	0.01
VED use (past 4 weeks)	465 (28.8)	138 (25.7)	156 (28.9)	171 (31.8)	1.24 (0.92–1.68)	0.16
Injection therapy (past 3 months)	454 (28.1)	124 (23.0)	148 (27.5)	182 (33.8)	1.58 (1.16–2.15)	0.004
PFMT engagement	146 (9.0)	38 (7.1)	48 (8.9)	60 (11.2)	1.73 (1.08–2.77)	0.02
Multi-modal treatment engagement						
Using ≥2 treatment modalities	583 (36.1)	162 (30.1)	194 (36.0)	227 (42.2)	1.56 (1.18–2.06)	0.002
Using ≥3 treatment modalities	194 (12.0)	48 (8.9)	65 (12.1)	81 (15.1)	1.89 (1.24–2.88)	0.003
Rehabilitation support utilization						
Has written rehabilitation plan	666 (41.2)	189 (35.1)	225 (41.7)	252 (46.8)	1.48 (1.13–1.94)	0.005
Received in-clinic training	630 (39.0)	181 (33.6)	212 (39.3)	237 (44.1)	1.38 (1.05–1.81)	0.02
Treatment adherence						
Good adherence (≥80%)	757 (46.9)	213 (39.6)	252 (46.8)	292 (54.3)	1.71 (1.30–2.25)	<0.001

OR indicates odds ratio; CI, confidence interval; PDE5i, phosphodiesterase type 5 inhibitors; VED, vacuum erection device; PFMT, pelvic floor muscle training. ^a^ Adjusted for age, education, marital status, nerve-sparing status, time since surgery, and preoperative erectile function. Statistical Analysis: Logistic regression models to examine odds of treatment utilization by health literacy level, controlling for clinical factors.

### 3.4 Attitudes and psychological factors

All attitudinal domains demonstrated significant associations with health literacy (Table 1). Treatment acceptability scores increased progressively across health literacy levels for both VED (3.4 ± 1.2 to 3.8 ± 1.0, *F* = 8.42, *p* < 0.001) and injection therapy (2.9 ± 1.4 to 3.5 ± 1.2, *F* = 12.67, *p* < 0.001). Self-efficacy showed particularly strong associations, with treatment self-efficacy scores ranging from 3.2 ± 1.2 in low health literacy to 3.8 ± 1.0 in high health literacy participants (*F* = 15.23, *p* < 0.001). Conversely, perceived barriers demonstrated inverse relationships with health literacy. Side effect concerns decreased from 3.5 ± 1.1 to 3.1 ± 1.1 (*F* = 7.94, *p* < 0.001), embarrassment from 3.0 ± 1.3 to 2.6 ± 1.1 (*F* = 6.47, *p* = 0.002), and cost barriers from 3.7 ± 1.1 to 3.1 ± 1.2 (*F* = 13.42, *p* < 0.001) across low to high health literacy levels.

### 3.5 Multivariable predictors and model performance

**Figure 3:**
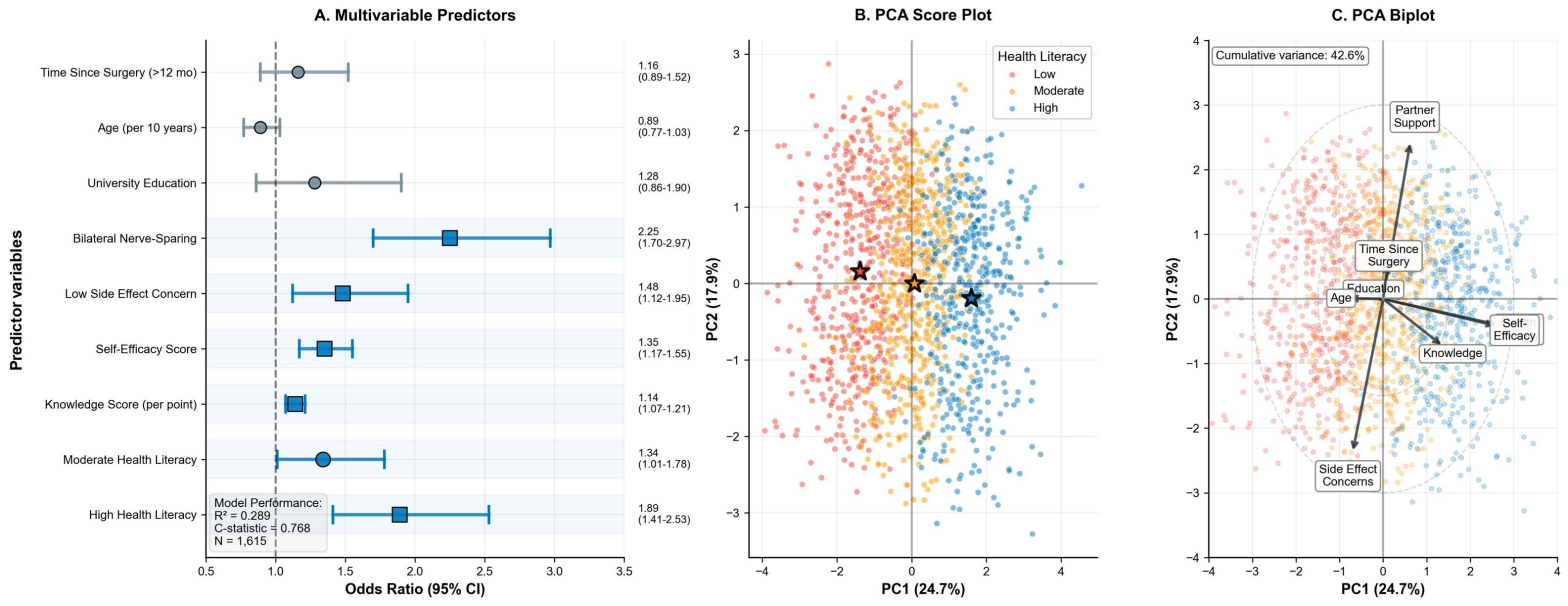
Predictive models and multidimensional analysis. **(A)** Forest plot of multivariable predictors for optimal sexual health recovery; points represent odds ratios (OR) with 95% confidence intervals (CI) relative to the null line (OR = 1.0), with model performance metrics inset. **(B)** PCA score plot visualizing patient distribution across the first two principal components, color-coded by health literacy. Star markers indicate group centroids; distinct separation highlights health literacy-driven clustering in rehabilitation profiles. **(C)** PCA biplot showing loading vectors for key variables; arrow length and direction indicate the strength of contribution and correlation structure relative to the principal components.

Hierarchical logistic regression modeling for optimal sexual health recovery practices (≥2 modalities plus ≥80% adherence) demonstrated substantial incremental predictive value across three models (Table 3 and Figure 3A). The final model (Model 3) achieved strong discrimination with a C-statistic of 0.768 and explained 28.9% of variance (Nagelkerke *R*^2^ = 0.289). Independent predictors in the final model included bilateral nerve-sparing status (OR 2.25, 95% CI: 1.70–2.97, *p* < 0.001), knowledge score per point increase (OR 1.14, 95% CI: 1.07–1.21, *p* < 0.001), self-efficacy score per point increase (OR 1.35, 95% CI: 1.17–1.55, *p* < 0.001), and low side effect concern (OR 1.48, 95% CI: 1.12–1.95, *p* = 0.005). Most notably, health literacy demonstrated strong independent effects with moderate health literacy showing an OR of 1.34 (95% CI: 1.01–1.78, *p* = 0.04) and high health literacy an OR of 1.89 (95% CI: 1.41–2.53, *p* < 0.001) compared to low health literacy. PCA visualizations further illustrated separation of rehabilitation profiles across health literacy groups and the contribution of key variables to the first two principal components (Figures 3B, C).

### 3.6 Mediation analysis

Structural equation modeling revealed that health literacy was associated with a significant indirect pathway between knowledge and practice implementation (Table 7), a pattern of associations consistent with statistical mediation. Cross-sectional data precludes causal inference; the observed associations may reflect true mediation (literacy enabling knowledge-to-behavior translation), reverse causation (rehabilitation experiences enhancing functional literacy), or shared unmeasured causes. While the temporal logic of the mediation model aligns with health behavior theory, causal directionality requires prospective or experimental confirmation. The total effect of knowledge on practice (*β* = 0.34, *p* < 0.001) was partially mediated through health literacy, with an indirect effect of 0.044 (95% CI: 0.024–0.071, *p* < 0.001). Health literacy mediated 31% of the knowledge-practice relationship (proportion mediated = 0.31, 95% CI: 0.18–0.49, *p* < 0.001), with both the Sobel test (z = 3.67, *p* < 0.001) and bias-corrected bootstrap confidence intervals confirming statistical significance.

**Table 7:** Mediation analysis of health literacy as mediator between knowledge and practice implementation.

Pathway	Coefficient (SE)	95% CI	*P-*value	Effect size^a^
Direct effects				
Knowledge → Practice (total effect)	0.142 (0.028)	0.087–0.197	<0.001	0.34
Knowledge → Practice (direct effect)	0.098 (0.031)	0.037–0.159	0.002	0.23
Knowledge → Health Literacy (a path)	0.187 (0.022)	0.144–0.230	<0.001	0.42
Health Literacy → Practice (b path)	0.234 (0.045)	0.146–0.322	<0.001	0.31
Indirect effects (Mediation)				
Knowledge → Health Literacy → Practice	0.044 (0.012)	0.024–0.071^b^	<0.001	0.11
Mediation statistics				
Proportion mediated	0.31 (0.08)	0.18–0.49^b^	<0.001	—
Sobel test statistic	3.67	—	<0.001	—
Model R^2^ (outcome)	0.284	—	—	—

SE indicates standard error; CI, confidence interval. ^a^ Standardized coefficients (β). ^b^ Bias-corrected bootstrap confidence intervals (5,000 iterations). Statistical Analysis: Structural equation modeling with bootstrapped confidence intervals to test mediation pathways (Sobel test and bias-corrected bootstrap).

### 3.7 Health literacy-stratified barriers and facilitators

Analysis of barriers and facilitators revealed systematic patterns across health literacy levels (Table 2). As literacy increased from low to high, significant treatment barriers declined, including concerns over side effects (44.1% vs. 28.1%; OR 0.51, 95% CI: 0.39–0.67, *p* < 0.001), embarrassment (35.1% vs. 20.8%; OR 0.49, 95% CI: 0.36–0.67, *p* < 0.001), and cost (49.1% vs. 32.3%; OR 0.50, 95% CI: 0.38–0.65, *p* < 0.001). Conversely, facilitators like strong partner support (68.2% vs. 78.6%; OR 1.72, 95% CI: 1.24–2.39, *p* = 0.01) and high self-efficacy (45.2% vs. 64.7%; OR 2.23, 95% CI: 1.69–2.95, *p* < 0.001) became more prevalent. Information-seeking behaviors showed the strongest associations, with dramatic increases in active information seeking (35.1% vs. 66.2%; OR 3.64, 95% CI: 2.74–4.84, *p* < 0.001), digital resource use (27.0% vs. 61.2%; OR 4.27, 95% CI: 3.15–5.78, *p* < 0.001), and support group participation (16.0% vs. 36.1%; OR 2.95, 95% CI: 2.12–4.11, *p* < 0.001).

### 3.8 Risk stratification and clinical decision support

Latent class analysis identified distinct patient phenotypes requiring tailored interventions (Table 8 and Figure 2B). These profiles comprised a high-risk group (22.0%) warranting immediate intervention—within which a 9.7% subset was characterized by low health literacy, poor knowledge (mean 5.2 ± 1.8), and minimal practice engagement (mean 1.1 ± 0.9)—together with moderate-risk (35.0%) and low-risk (32.4%) groups appropriate for targeted and maintenance support, respectively. The risk stratification model showed good predictive validity (AUC = 0.78) for identifying patients needing intensive support (Figure 2A). The corresponding intervention pathway model (Figure 2D) illustrates a tiered approach, assigning low health literacy patients to intensive education, moderate literacy patients to counseling plus education, and high literacy patients to standard care, with practice implementation contingent on intervention effectiveness.

**Table 8:** Clinical risk stratification and intervention priority matrix.

**Patient profile** ^**a**^	***n* (%)**	**Knowledge score** **Mean (SD)**	**Practice engagement** **score Mean (SD)**	**Intervention priority**	**Recommended approach**
High-risk profiles (immediate intervention)					
Low HL + Low knowledge + Poor practice	156 (9.7)	5.2 (1.8)	1.1 (0.9)	Priority 1	Intensive individualized support
Low HL + Moderate knowledge + Poor practice	198 (12.3)	7.1 (1.5)	1.4 (1.0)	Priority 1	Skills-based training
Moderate-risk profiles (targeted intervention)					
Moderate HL + Good Knowledge + Poor practice	143 (8.9)	8.7 (1.4)	1.6 (1.1)	Priority 2	Barrier-focused counseling
High HL + Moderate knowledge + Moderate practice	187 (11.6)	7.8 (1.7)	2.3 (1.0)	Priority 2	Knowledge enhancement
Moderate HL + Moderate knowledge + Moderate practice	234 (14.5)	7.6 (1.6)	2.1 (0.9)	Priority 2	Standard care enhancement
Low-risk profiles (Monitoring/Maintenance)					
High HL + High knowledge + Good practice	289 (17.9)	9.4 (1.2)	3.2 (0.8)	Priority 3	Maintenance support
High HL + Good knowledge + Good practice	234 (14.5)	8.8 (1.3)	3.0 (0.9)	Priority 3	Peer support role
Mixed profiles (others)	174 (10.8)	7.4 (2.1)	2.0 (1.2)	Priority 2–3	Individualized assessment

HL indicates health literacy. ^a^ Patient profiles derived from latent class analysis using health literacy, knowledge, and practice engagement scores. Knowledge categories: Low (<6), Moderate (6–8), High (>8). Practice categories: Poor (<2), Moderate (2–3), Good (>3). Statistical Analysis: Latent class analysis to identify distinct patient phenotypes requiring tailored interventions, with clinical validation using ROC analysis (AUC = 0.78).

## 4 Discussion

The present study provides compelling evidence of the pivotal role HL plays in shaping KAP related to sexual health recovery among Chinese men following radical RP for prostate cancer, as well as their intimate partners. In a large single-center cohort, HL displayed a clear dose–response gradient across all major endpoints—higher literacy was associated with greater rehabilitation knowledge, higher treatment acceptability and self-efficacy, more frequent and multimodal engagement (PDE5i, VED, intracavernosal injections, and PFMT), and significantly better adherence (≥80%). Importantly, multivariable models with strong discrimination showed that HL retained independent predictive value after adjustment for nerve-sparing status, knowledge, and attitudinal factors, while mediation analysis quantified that approximately one-third of the knowledge→practice association operated through HL. Complemented by literacy-stratified barrier and facilitator profiles and a latent-class risk algorithm with good predictive validity, these findings reposition HL from a background covariate to a central, modifiable lever for implementing evidence-based sexual rehabilitation in routine Chinese urology practice.

In relation to established modalities, the observed literacy-graded increases in the use of PDE5i, VED, intracavernosal injection therapy, and PFMT accord with syntheses indicating that pharmacologic and device-based strategies improve erectile function during active use, whereas effects on spontaneous recovery are variable and protocol-dependent (9, 38, 39). Contemporary meta-analytic work supports PDE5i efficacy after nerve-sparing RP with improvements in International Index of Erectile Function (IIEF) domains compared with placebo, particularly under regular-dosing schedules rather than on-demand regimens (40, 41). Likewise, recent scoping and systematic reviews suggest that early, protocolized VED use can support penile hemodynamics and expedite functional recovery, and that combined strategies (e.g., PDE5i + VED) may augment outcomes for selected men (9). Adjunctive modalities—such as low-intensity shock wave therapy (LI-SWT) layered on daily tadalafil—have shown mixed but promising signals in comparative cohorts, reinforcing the premise that multimodal, scheduled care may outperform monotherapy in real-world settings (42). Against this background, the present gradients in both utilization and adherence by HL indicate that even efficacious strategies will underperform when patients struggle to access, understand, or execute complex regimens.

The magnitude of HL effects observed here is clinically meaningful when considered alongside surgical determinants. Bilateral nerve-sparing status remained a strong predictor of implementation success, consonant with contemporary evaluations linking nerve preservation to superior functional recovery potential (43–45). However, the persistence of HL effects after controlling for nerve sparing suggests that literacy-sensitive processes—comprehension of rehabilitation rationales, correct device technique, management of side effects, and help-seeking when difficulties arise—constitute a parallel mechanism influencing outcomes. Stated differently, anatomical preservation creates capacity for recovery, whereas HL-dependent behaviors actualize that capacity through consistent practice and timely troubleshooting. This behavioral pathway is further supported by the independent contributions of self-efficacy and low side-effect concern in the final model, aligning with evidence that cognitive-behavioral constructs predict sexual adjustment and that education-enhanced survivorship programs can improve self-efficacy and symptom management (46–48).

Mediation analysis extends prior qualitative and theoretical work by quantifying a specific pathway through which knowledge may be converted to action. While cross-sectional data preclude definitive causal claims, the finding that HL statistically mediates 31% of the knowledge→practice association is consistent with the hypothesis that knowledge alone is insufficient unless patients can access, interpret, and apply information within their sociocultural and healthcare contexts. Alternative explanations—such as reverse causation (successful rehabilitation experiences enhancing functional health literacy) or unmeasured common causes (e.g., cognitive function, educational quality) driving both literacy and adherence—cannot be excluded and warrant prospective investigation. Recent oncology literature has documented associations between HL and survival, quality of life, and self-management behaviors across tumor types (16, 49, 50). In prostate cancer specifically, prospective data indicate that baseline HL and eHealth literacy are positively associated with postoperative quality of life after RP, highlighting the need to incorporate literacy assessment into routine surgical counseling and survivorship planning (35). The current results provide suggestive mechanistic plausibility for these associations by showing that HL correlates with adherence via improved self-efficacy and reduced attitudinal barriers, thereby potentially facilitating transformation of information into sustained, protocol-concordant practice.

Attitudinal gradients observed across HL strata—higher acceptability for VED and injections, greater self-efficacy, and lower concerns regarding adverse effects, embarrassment, and cost—map onto determinants known to influence rehabilitation fidelity. Regarding the dyadic nature of sexual recovery, several important caveats temper the dyadic interpretation of our findings. Partner data in this study primarily enriched measurement of the partner support construct rather than enabling independent partner-level analyses or rigorous testing of dyadic interdependence models. With partner participation at 74.5%, we lacked partner-reported data for one-quarter of the sample, and while missingness appeared random with respect to patient characteristics, partners who accompanied patients to clinic visits likely represent more engaged, supportive relationships. This selection bias may have inflated partner support estimates and obscured relationship strain or partner resistance as barriers to rehabilitation, particularly in low-literacy dyads where communication challenges may be most pronounced. While higher patient literacy associated with stronger support (Table 2), cross-sectional data and our patient-level analytic approach (vs. Actor-Partner Interdependence Models requiring complete dyads) preclude conclusions about causal directionality, reciprocal influences, or independent partner literacy effects. Patient literacy may influence partner engagement, or supportive partnerships may facilitate literacy development—prospective dyadic studies are needed to disentangle these pathways. Notably, partner health literacy itself (measured in the 74.5% of dyads with participating partners) showed moderate correlation with patient health literacy (*ρ* = 0.34), raising important questions about whether dyadic literacy concordance amplifies or mitigates individual literacy effects. Future dyadic studies should model actor-partner interdependence to parse these pathways and test whether couple-based interventions differentially benefit literacy-discordant versus concordant dyads. These patterns also intersect with the dyadic nature of sexual recovery. Contemporary randomized and quasi-experimental studies of couple-based or psychosexual programs have reported consistent relational benefits, earlier resumption of intimacy, and improved communication, albeit with mixed effects on global quality-of-life endpoints (51–53). The present study adds that higher HL is associated with stronger partner support and more active information-seeking, suggesting that literacy-responsive dyadic curricula could amplify technical rehabilitation (PDE5i, VED, injections, PFMT) by enhancing confidence, normalizing sexual discourse, and addressing stigma. Conversely, in lower-HL days, embarrassment and side-effect concerns may persist without targeted counseling and skills-based training, undermining adherence even when prescriptions are provided.

The digitalization of survivorship care offers both opportunities and challenges that are tightly linked to HL and, importantly, to the related but distinct construct of eHealth literacy. Web-based and nurse-led telehealth supports (e.g., the TrueNTH platform and the Sexual Health and Rehabilitation eClinic) have demonstrated feasibility and acceptability. In some trials, these interventions have accelerated the resumption of sexual activity. However, effects on sexual satisfaction or quality of life are inconsistent, and implementation barriers remain (53–55). A recent systematic review suggests that men with prostate cancer and their carers often exhibit novice-level eHealth literacy, reported limited use of contemporary digital tools, which may blunt the impact of unguided online interventions (56). Theoretically, eHealth literacy builds upon foundational general health literacy. It requires not only the ability to understand health content but also skills to navigate digital interfaces, evaluate online information credibility, protect privacy, and engage with interactive features.

Our finding that general HL strongly predicted digital resource use (OR 4.27 for high vs. low HL; Table 2) supports this hierarchical relationship and suggests that individuals with limited general HL face compounded barriers when rehabilitation moves to digital platforms. Conversely, high general HL may not automatically confer eHealth competence, particularly among older adults with limited prior digital exposure. This mismatch has critical implications for telehealth equity: deploying web-based rehabilitation platforms without addressing foundational general HL and providing digital literacy scaffolding (e.g., tutorial videos, live onboarding sessions, tech support hotlines) risks widening disparities rather than bridging them. The strong HL gradient in digital information-seeking observed here indicates that telehealth initiatives should incorporate literacy scaffolding (plain-language microlearning, step-by-step device tutorials, teach-back verification, and moderated forums), particularly for older adults and those with minimal prior digital engagement. When designed, digital platforms can deliver symptom tracking, tailored feedback, and timely troubleshooting that support adherence at scale (55–57).

Contextualizing these findings within China underscores their health-system relevance. China recorded an estimated 4.82 million new cancer cases in 2022, with an aging demographic concentrating the burden among older adults who typically have lower HL (31, 58). National surveillance and narrative syntheses document growth in HL since 2008 but still indicate that fewer than one in three adults have adequate HL, with particularly low levels in rural and older populations (31). Concurrently, Chinese qualitative studies continue to identify stigma surrounding sexual health discourse, unmet informational needs, and partner strain among prostate cancer survivors, all of which likely depress engagement with sexual rehabilitation (28, 36). In this context, the present literacy-stratified disparities in multimodal engagement and adherence delineate a critical and modifiable target for service redesign. Moreover, emerging randomized data—including scheduled daily tadalafil combined with VED—indicate that protocolized, combination strategies can outperform monotherapy after nerve-sparing RP, reinforcing the need to translate evidence into accessible, literacy-responsive pathways (9, 30, 36, 59).

From an implementation standpoint, the latent class analysis provides a pragmatic potential template for stepped care by resolving heterogeneous couples into phenotypes that differ in literacy, knowledge, and practice. The model’s predictive validity (AUC = 0.78) suggests that routine stratification could prioritize resources: intensive education and hands-on training (e.g., in-clinic VED/injection technique with checklists) for high-need, low-HL dyads; targeted counseling plus simplified written plans for intermediate profiles; and maintenance support with remote monitoring for high-HL, high-practice groups. However, cross-sectional design means these profiles represent observed associations rather than validated prognostic scores; prospective validation in independent cohorts is essential before clinical deployment. Additionally, whether literacy-tailored interventions reduce disparities and improve functional outcomes vs. simply identifying high-risk patients—requires randomized trials. Such an approach parallels multi-institutional survivorship programs and aligns with evidence that exercise and psychosexual education can augment sexual outcomes when integrated into comprehensive care (30, 59). Importantly, the present data move beyond modality selection to emphasize the literacy-responsive delivery of those modalities, thereby addressing a concrete translational gap. The translation of these findings into diverse clinical settings warrants careful consideration of institutional capacity and resource availability. Our tertiary center context—with established urology survivorship clinics, on-site specialized nurses, routine access to all four rehabilitation modalities, and optional psychosexual counseling—represents an implementation-ready environment that may not exist in community hospitals or rural clinics where prostate cancer care is increasingly delivered. In such settings, literacy-tailored interventions must be adapted to local constraints: simplified written materials and teach-back protocols can be universally applied regardless of device availability; telehealth-delivered counseling (if digital infrastructure permits) can extend specialist support to remote patients; and partnerships with primary care or general oncology services can ensure continuity when specialized urology follow-up is infrequent. Critically, low-literacy patients in under-resourced settings face compounded barriers—both the intrinsic literacy-related challenges documented here and the extrinsic access barriers that disproportionately affect such communities. Implementation research should therefore test whether tiered, literacy-responsive interventions reduce disparities in high-resource contexts while simultaneously evaluating strategies (e.g., community health worker-delivered education, peer navigation programs) that address both literacy and access barriers in lower-resource settings.

Future research should prioritize multi-center randomized trials testing step, literacy-tailored rehabilitation packages against usual care, focusing on adherence, functional outcomes, and cost-effectiveness. Factorial designs could isolate active intervention components, while prospective analyses should test if changes in health literacy and self-efficacy mediate improved outcomes. Hybrid effectiveness-implementation studies are needed to assess the fidelity, reach, and sustainability of these programs within existing health systems. Finally, digital innovations like adaptive messaging should be evaluated for their capacity to support adherence in low-literacy populations without exacerbating digital inequities. While our findings identify health literacy as a strong correlate of rehabilitation practices, critical implementation evidence gaps remain. This cross-sectional study cannot determine whether: (1) literacy-tailored materials improve comprehension and adherence vs. standard materials; (2) intensive training for low-literacy patients yields functional gains beyond device access; (3) tiered delivery is cost-effective vs. universal high-intensity support; or (4) improved adherence translates to better erectile function and quality of life. These questions require randomized trials with literacy-stratified vs. uniform interventions, functional outcomes, and cost-effectiveness analysis. Until such evidence exists, our risk stratification framework should be viewed as hypothesis-generating rather than a validated clinical decision tool.

The dramatic health literacy gradient in digital resource use (OR 4.27 for high vs. low HL) must be understood within China’s evolving digital healthcare landscape and persistent digital divide. National data indicate that while overall internet penetration reached 73% in 2022, substantial disparities exist by age and geography: among adults ≥60 years, internet usage is 43% compared to 98% among those aged 18–39 years, and rural residents show 17 percentage points lower adoption than urban populations. Smartphone ownership follows similar patterns, with only 52% of rural older adults possessing smartphones capable of accessing health apps. Moreover, digital health literacy—comprising not just access but also navigation skills, information evaluation, and privacy management—remains limited even among internet users, with older adults reporting low confidence in telehealth platforms and concerns about data security. These structural barriers intersect with general health literacy in multiplicative fashion: low-HL patients who are also older, rural-dwelling, and digitally inexperienced face compounded obstacles when rehabilitation shifts to web-based platforms. Our finding that only 27% of low-literacy patients used digital health resources (vs. 61% of high-literacy patients) likely reflects both limited general HL (difficulty understanding online health content) and limited digital access/skills. Consequently, telehealth-based rehabilitation programs—while scalable and cost-effective in principle—risk exacerbating disparities unless accompanied by: (1) offline alternatives (telephone coaching, printed materials with visual aids); (2) digital literacy onboarding (in-person tutorials, simplified interfaces); (3) infrastructure support (subsidized smartphones, public Wi-Fi access at clinics); and (4) family/caregiver engagement (training adult children to facilitate parents’ platform use). In China’s tiered healthcare system, where tertiary urban hospitals increasingly adopt digital survivorship tools while rural township clinics lack basic internet connectivity, literacy-responsive implementation must account for profound variation in digital readiness across care settings.

The translation of these findings requires careful consideration of institutional capacity. Our tertiary center—with specialized nurses, routine access to all four modalities, and optional psychosexual counseling—represents an implementation-ready environment uncommon in community or rural clinics. In such settings, literacy-tailored interventions must adapt to constraints: simplified materials and teach-back protocols are universally applicable; telehealth-delivered counseling can extend specialist support where digital infrastructure permits; primary care partnerships ensure continuity when urology follow-up is infrequent. Critically, low-literacy patients in under-resourced settings face compounded barriers—both intrinsic literacy challenges and extrinsic access barriers. Implementation research should test whether tiered interventions reduce disparities in high-resource contexts while evaluating strategies (community health workers, peer navigation) addressing both literacy and access barriers in lower-resource settings.

Several limitations merit consideration. First, the single-center, cross-sectional design precludes causal inference regarding health literacy effects and limits generalizability. Recruitment from a tertiary center with established infrastructure likely represents a best-case scenario; observed literacy gradients may be attenuated in resource-constrained settings (where structural barriers impede utilization regardless of literacy) or magnified in settings lacking systematic protocols (where high-literacy patients compensate via self-directed seeking while low-literacy patients receive minimal guidance). Findings are most generalizable to tertiary clinics with multimodal rehabilitation programs; translation to community/rural practices requires adaptation to local capacity, staffing, and reimbursement contexts. While literacy-rehabilitation associations likely extend beyond Chinese settings—consistent with Western cohort data—context-specific factors (cultural attitudes toward sexual disclosure, healthcare organization, digital infrastructure) may moderate effect magnitudes. Replication across diverse geographic and cultural contexts is warranted to establish cross-cultural validity of observed mediation pathways and risk stratification.

Second, self-reported adherence may overestimate true rates despite structured recall windows and logic checks; objective measures (pharmacy refills, device logs) would strengthen estimates. Third, residual confounding by unmeasured variables (socioeconomic status, cognitive function, partner health literacy) cannot be excluded. Fourth, while the HLS19-Q12 demonstrated good internal consistency (*α* = 0.87), broader validation against functional literacy tasks in older Chinese cohorts remains a priority. Fifth, incomplete partner participation (74.5%) may have introduced selection bias toward engaged, supportive relationships, potentially inflating partner support estimates and obscuring relationship strain as a barrier. Sixth, tertile categorization of health literacy is sample-dependent and may not generalize to populations with markedly different literacy distributions. Finally, the hypothesis-generating risk stratification model requires prospective validation in independent cohorts before clinical deployment, and whether literacy-tailored interventions improve functional outcomes (vs. simply identifying high-risk patients) remains an empirical question requiring randomized trials.

Notwithstanding these limitations, findings have actionable implications for survivorship care redesign: (1) integrate brief health literacy screening (e.g., HLS19-Q12) into routine urology consultations to enable risk stratification; (2) standardize plain-language written plans with pictograms and teach-back verification; (3) pair early multimodal rehabilitation initiation (daily PDE5i, adjunct VED, structured PFMT) with skills training and follow-up coaching for low-literacy patients; (4) design telehealth platforms with digital literacy scaffolding (tutorials, simplified interfaces, tech support) to avoid exacerbating disparities; and (5) systematically address attitudinal barriers (embarrassment, side-effect concerns, costs) through anticipatory guidance and financial counseling referrals.

## 5 Conclusion

This study identifies health literacy as a fundamental correlate of sexual health recovery following RP, with statistical associations consistent with mediation of the knowledge-practice relationship and revealing systematic disparities in treatment utilization, adherence, and perceived barriers across health literacy levels. While cross-sectional design precludes causal inference, observed patterns are consistent with theoretical frameworks positing that patients’ capacity to find, understand, and apply health information shapes rehabilitation engagement beyond simple knowledge acquisition. Our risk stratification model enables hypothesis-generating identification of distinct patient phenotypes that may benefit from tailored intervention strategies, providing a potential framework for implementing precision rehabilitation care that matches support intensity to patient capabilities. These findings demonstrate that substantial literacy-stratified disparities in rehabilitation engagement exist—with adherence rates differing by 14.7 percentage points and digital resource use varying fourfold across literacy levels—warranting prospective trials of routine health literacy assessment and tiered intervention strategies to determine whether such approaches actually reduce inequities and improve functional outcomes. Until randomized implementation trials provide causal evidence, our risk stratification algorithm should be viewed as hypothesis-generating rather than a validated clinical decision tool.

## Data Availability

The raw data supporting the conclusions of this article will be made available by the authors, without undue reservation.
