# Supplementary material for: Health literacy correlates, barriers, and adherence in post-prostatectomy sexual rehabilitation: cross-sectional findings from China
Source: Front Public Health. 2026 Mar 20;14:1725144. doi: 10.3389/fpubh.2026.1725144 (PMC13047086; doi:10.3389/fpubh.2026.1725144)
Supplement: Supplementary file 1 [file Table_1.docx]

**Supplemental Table 1. Operationalization of Tiered Sexual Rehabilitation Interventions by Health Literacy Level**

| Intervention Tier | Target Population | Core Components | Delivery Mode | Estimated Contact Time | Key Implementation Considerations |
| --- | --- | --- | --- | --- | --- |
| Tier 1: Intensive Support | Low health literacy + low knowledge + poor practice (Priority 1: 22% of cohort) | • Plain-language written rehabilitation plan with pictograms • In-person device demonstrations (VED, injection technique) with return demonstration • Teach-back verification of key concepts • Weekly telephone coaching (weeks 1-4) • Troubleshooting hotline access | Initial in-person session (60-90 min) + 4 weekly follow-up calls (15-20 min each) + as-needed support | ~3 hours total over first month | • Requires specialized nurse or educator training • May need interpreter services • Partner attendance encouraged • Simplified side-effect management checklist |
| Tier 2: Targeted Support | Moderate health literacy + mixed knowledge/practice profiles (Priority 2: 35% of cohort) | • Standardized written plan (8th grade reading level) • Barrier-focused counseling session • Brief device training if prescribed • Biweekly text message reminders • Optional telehealth check-in at 4 weeks | Initial in-person session (30-45 min) + optional telehealth follow-up (20 min) | ~1 hour total | • Standardized materials reduce prep time • Telehealth option increases scalability • Automated reminders via SMS platform |
| Tier 3: Maintenance Support | High health literacy + good knowledge + active practice (Priority 3: 32% of cohort) | • Standard written rehabilitation plan • Access to online patient portal with educational videos • Peer support group invitation • Routine clinic follow-up only | Standard clinic encounter + self-directed digital resources | ~15-20 min (standard visit) | • Minimal additional resources required • May serve as peer mentors for Tier 1 patients • Digital platform maintenance needed |

Contact time estimates exclude routine clinical follow-up visits. Resource requirements will vary by institutional capacity; in under-resourced settings, Tier 1 interventions may be adapted using trained community health workers or group education formats. Partner participation encouraged across all tiers. Intervention assignment based on baseline health literacy assessment (HLS19-Q12) plus brief knowledge screening; reassessment at 3-6 months allows tier adjustment based on progress.
